# The *Staphylococcus aureus* ArlS Kinase Inhibitor Tilmicosin Has Potent Anti-Biofilm Activity in Both Static and Flow Conditions

**DOI:** 10.3390/microorganisms12020256

**Published:** 2024-01-25

**Authors:** Zihui Wang, Haoran Wang, Jinna Bai, Shen Cai, Di Qu, Youhua Xie, Yang Wu

**Affiliations:** Key Laboratory of Medical Molecular Virology (MOE/NHC/CAMS), Shanghai Frontiers Science Center of Pathogenic Microorganisms and Infection, School of Basic Medical Sciences, Shanghai Medical College, Fudan University, Shanghai 200032, Chinacaishen@fudan.edu.cn (S.C.);

**Keywords:** *Staphylococcus aureus*, MRSA, biofilm, inhibitor, two-component system

## Abstract

*Staphylococcus aureus* can form biofilms on biotic surfaces or implanted materials, leading to biofilm-associated diseases in humans and animals that are refractory to conventional antibiotic treatment. Recent studies indicate that the unique ArlRS regulatory system in *S. aureus* is a promising target for screening inhibitors that may eradicate formed biofilms, retard virulence and break antimicrobial resistance. In this study, by screening in the library of FDA-approved drugs, tilmicosin was found to inhibit ArlS histidine kinase activity (IC_50_ = 1.09 μM). By constructing a promoter-fluorescence reporter system, we found that tilmicosin at a concentration of 0.75 μM or 1.5 μM displayed strong inhibition on the expression of the ArlRS regulon genes *spx* and *mgrA* in the *S. aureus* USA300 strain. Microplate assay and confocal laser scanning microscopy showed that tilmicosin at a sub-minimal inhibitory concentration (MIC) had a potent inhibitory effect on biofilms formed by multiple *S. aureus* strains and a strong biofilm-forming strain of *S. epidermidis*. In addition, tilmicosin at three-fold of MIC disrupted USA300 mature biofilms and had a strong bactericidal effect on embedded bacteria. Furthermore, in a BioFlux flow biofilm assay, tilmicosin showed potent anti-biofilm activity and synergized with oxacillin against USA300.

## 1. Introduction

*Staphylococcus aureus* is an important pathogen causing localized and systemic infections in both humans and animals, which are associated with high morbidity [1]. The methicillin-resistant *S. aureus* (MRSA) strains are spreading globally and regionally, not only causing community-acquired and nosocomial infections but also becoming more resistant to a broad spectrum of commonly used antibiotics [2,3], such as β-lactams, aminoglycosides and macrolides. Furthermore, the increasing MRSA infection and colonization in both food-chain and companion animals show that MRSA is also an important zoonotic and veterinary pathogen [4].

*S. aureus* has the ability to produce biofilms both in vitro and in vivo (e.g., wounds and organs in humans and animals, implanted medical devices, etc.), which enhances its resistance and tolerance to antibiotic treatment and makes this pathogen a major cause of refractory biofilm-related diseases [5,6,7]. It has been estimated that over 80% of human infectious diseases are related to bacterial biofilms [8]. Moreover, approximately 60% of nosocomial infections are associated with bacterial biofilms formed on medical implants [9,10,11,12,13]. Furthermore, there are reports that about 61% of biofilm infections in humans have a zoonotic origin [14], and bacterial biofilms are closely related to animal diseases, such as mastitis, wound infection and periodontal disease [15]. Thus, the importance of *S. aureus* biofilm in clinical medicine and veterinary medicine should not be underscored [16].

The major concern of biofilm-related infections is that the bacterial cells embedded in biofilm exhibit inherent resistance to antimicrobial agents [17]. Previous studies have already shown that biofilm bacteria are 10–1000 times more resistant to antibiotic killing and act as a source of persistent and chronic infections [18,19]. The underlying mechanisms are very complicated, including the slow penetration of drugs through the extracellular polymeric substance (EPS), the reduced susceptibility of bacteria cells inside biofilm matrix due to a low metabolic state (e.g., persisters) [20], the altered micro-environment in different layers of biofilms (e.g., pH, oxygen), the changed transcription profiles of bacteria under stress [6,7,21], the facilitated horizontal gene transfer among different bacterial species, etc. [22,23,24]. Therefore, there is an urgent need to develop new antimicrobial agents and discover already-in-use drugs with anti-biofilm activities to overcome the ineffectiveness of conventional antibacterials and to fight against staphylococcal biofilm-related diseases [25,26,27].

Bacterial two-component signal transduction systems (TCSTs) play a key role in sensing various stimulus signals and making corresponding responses to help bacteria cells adapt better to environmental stress [28,29,30,31]. Moreover, TCSTs have no human homologs and thus have been considered potential targets for antimicrobial therapy [32]. Among the 16–17 TCSTs in *S. aureus,* ArlRS (autolysis-related locus) is a unique one with a global regulatory effect on *S. aureus* virulence, autolysis, slime layer formation, cell aggregation and biofilm development in vitro [33,34,35,36,37,38,39]. Our group previously reported *arlRS* deficiency or deletion in *S. epidermidis* abolished biofilm production [40,41]. A similar influence of *arlRS* inactivation on *S. aureus* biofilm development has also been reported in vitro and in vivo [42,43]. Furthermore, our group demonstrates that ArlRS directly regulates the transcription of a redox regulator *spx* and is vital for modulating oxacillin susceptibility [44]. These findings make ArlRS a promising target for screening anti-biofilm compounds and antibiotic resistance breaker drugs [45,46].

A typical TCST is composed of two proteins: a histidine kinase (HK), which is a transmembrane protein, and a response regulator (RR), which is a cytoplasmic DNA binding protein. HK is considered a promising drug target [47,48,49] because its autophosphorylation is mediated via the conserved catalytic and ATP-binding (CA) domain that contains a typical substrate binding pocket, which may be suitable for drug screening [32]. In this work, we screened the FDA-approved drug library for ArlS HK inhibitors and found that tilmicosin has a strong inhibitory effect on ArlS kinase activity. Tilmicosin is a macrolide that is active against Gram-positive bacteria by inhibiting bacterial protein synthesis. It can bind to the bacterial 50S subunit of the ribosome, thereby blocking polypeptide elongation and releasing [50]. The effect of tilmicosin on the biofilm formation of multiple staphylococcal strains was investigated in this study, which indicates that it has an additional antibacterial mechanism.

## 2. Materials and Methods

### 2.1. Strains and Plasmids

The MRSA strains USA300 FPR3757, USA300 TCH1516 and USA500 2395 (GenBank Accession Number: NC_007793, NC_010079 and CP007499, respectively) [51,52,53] and the methicillin-sensitive *S. aureus* Newman and *S. epidermidis* 1457 (GenBank Accession Number: NC_009641 and NZ_CP020463.1, respectively) [54,55] were used as wild-type strains in this study. The MRSA strains 234 and 15,098 were biofilm-positive clinical isolates [44]. The TCH1516Δ*arlRS*, USA500Δ*arlRS* and 1457Δ*arlRS* were previously constructed in our laboratory [40,44], which were corresponding *arlRS* gene-knockout mutants of the USA300 TCH1516, USA500 2395 and 1457 strains. The *arlRSc* (TCH1516) and *arlRSc* (1457) were corresponding *arlRS* complementation strains of the TCH1516Δ*arlRS* and 1457Δ*arlRS.* The bacterial strains that were constructed in this work are listed in Table 1.

The pCM29, pRB475, pCN51 and pKOR-1 are *Escherichia coli*-*S. aureus* shuttle vectors [44,56,57]. pCM29 is used to build a promoter-GFP reporter system. pRB475 and pCN51 are used to construct *arlRS* complementation plasmids pCN-*arlRS* and pRB-*arlRS* [58,59]. The temperature-sensitive plasmid pKOR1 is used for gene knockout in staphylococci [60]. pETMG is used for protein expression [61]. The plasmids constructed in this work are listed in Table 1.

### 2.2. Growth Media and Chemical Agents

Tryptic soy broth (Oxoid, UK) was used for the proliferation of *S. aureus* and *S. epidermidis* strains. TSB medium with 1% glucose (TSBG) was used for staphylococcal biofilm formation [59,62]. Luria–Bertani (LB, Oxoid, UK) medium was used for *E. coli* cultivation. Calcium-adjusted Mueller–Hinton Broth (CAMHB, Oxoid, UK) was chosen for the antimicrobial susceptibility test (AST) [10,63,64,65]. The antibiotics ampicillin (100 µg/mL), kanamycin (50 µg/mL), chloramphenicol (10 µg/mL) and erythromycin (10 µg/mL) were used for selection of the constructed bacterial strains. Tilmicosin and oxacillin were purchased from MedChemExpress China and Sangon Biotech (Shanghai, China) Co., Ltd., respectively.

### 2.3. Construction of the arlRS Deletion Mutants

The *arlRS* locus in the Newman strain (locus tag: NWMN_1327 and NWMN_1328) and the USA300 FPR3757 strain (locus tag: SAUSA300_1307 and SAUSA300_1308) were deleted by homologous recombination, as previously described [60], generating NewmanΔ*arlRS* and FPR3757Δ*arlRS,* respectively [12]. Afterward, the plasmids harboring *arlRS*, pRB-500*arlRS* and pRB-NM*arlRS* were built and transformed into the gene-knockout mutants by electroporation for *arlRS* complementation [66,67].

### 2.4. Semi-Quantitative Detection of Static Biofilms Formation

Biofilm-forming abilities of the staphylococcal strains on polymeric surfaces were assessed by a microplate assay [25]. The overnight bacterial cultures were 1:200 diluted in fresh TSBG, added into tissue-culture-treated 96-well microplates (Nunc, Denmark) and incubated for 24 h at 37 °C [7]. To detect the activities of tilmicosin against *S. aureus* and *S. epidermidis* biofilms formation in static condition, tilmicosin was added to TSBG culture medium at a 1/4 of MIC (0.78 μΜ or 0.67 μg/mL) to minimize its effect on bacterial growth. Several biofilm-producing MRSA strains, including USA300 TCH1516, FPR3757, USA500; two clinic isolates, 234 and 15,098; together with a strong biofilm-forming *S. epidermidis* strain 1457, were cultured in TSBG overnight, then 1:100 diluted with TSBG supplemented with tilmicosin in the wells of microtiter plates. Biofilms in the wells were fixed with 99% methanol, stained with 1% violet crystal and then dissolved with 10% acetic acid. Biofilm formation by each strain was assessed at OD_570_ in a plate reader (Victor X5, PerkinElmer, Boston, MA, USA).

### 2.5. Purification of the Catalytic Domain of ArlS (ArlSHK)

To obtain the intracellular region of *S. aureus* ArlS with histidine kinase activity, the expression plasmid (pETMG-*arlSHK*) was constructed and transformed into *E. coli* BL21 (DE3) [68,69,70]. The bacterium was cultured in LB for 4 h at 37 °C, and then 0.4 mM isopropyl β-D-1-thiogalactopyranoside was added for overnight induction at 25 °C. Afterward, the cells were washed with lysis buffer and sonicated at 4 °C. The recombinant ArlSHK consisted of a HisKA and a HATPase_c domain was fused with a GB1-tag (IgG domain B1 of Protein G) and purified using a Qiagen Ni-NTA column by affinity chromatography.

### 2.6. ATPase Assay for Screening ArlSHK Kinase Activity Inhibitors

To screen ArlSHK ATPase activity inhibitors, the Promega Kinase-Glo(R) Luminescent Kinase Assay was used as previously described [44,71,72,73]. The optimized condition for reactions was as follows: 2 μg purified ArlSHK, 3.5 μM ATP, 30 min incubation at room temperature. The luminescence was measured with a Victor X5 Reader. To detect the half-maximal inhibitory concentration (IC_50_) of the inhibitor, a series of dilutions of the compound were pre-incubated with ArlSHK, and then ATP was added. The IC_50_ value was calculated by using the sigmoidal fit module of the Origin 9.0 software (OriginLab).

### 2.7. Promoter-Florescence Reporter Assay

To investigate the effect of tilmicosin on ArlRS regulation in *S. aureus*, the previously reported *mgrA* P2 promoter and *spx* P2 promoter from the FPR3757 genome were chosen for constructing the promoter-florescence reporter system in the USA300 FPR3757 strain and its Δ*arlRS* mutant, as previously described [44]. Tilmicosin was added to the mid-exponential phase culture of the reporter strains of FPR3757 at a concentration of 0.75 μM or 1.5 μM. The DMSO treatment served as a negative control, and the reporter strains of Δ*arlRS* were used as low *spx* and *mgrA* expression controls.

### 2.8. Visualization of Three-Dimensional Structure of Biofilms by Confocal Laser Scanning Microscopy (CLSM)

Bacteria were inoculated into the glass-bottomed dishes (FluoroDish, WPI, Sarasota, FL, USA) containing 2 mL of TBSG. After incubation for a certain time, the unattached cells were carefully removed, and then the biofilms formed on glass were gently washed with normal saline and subsequently stained with SYTO9 and propidium iodide (PI, Molecular Probes, Eugene, OR, USA). A TCS SP8 microscope (Leica, Heidelberg, Germany) was used to acquire fluorescent images, determine biofilm thickness and analyze fluorescence intensities.

The strain USA300 FPR3757 was selected to investigate the impact of drugs on its biofilm formation. After live/dead staining, the three-dimensional view of the biofilm was generated with the IMARIS 7.0 software (Bitplane, Zurich, Switzerland).

### 2.9. Antimicrobial Susceptibility Test

The broth microdilution method was performed to detect minimal inhibitory concentrations (MIC) of the tilmicosin and oxacillin in *S. aureus* strains, as previously described [74].

The checkerboard method was used to determine if drugs are synergistic for inhibiting *S. aureus* proliferation (synergism is defined as a fractional inhibitory concentration index (FICI) ≤ 0.5 [75]).

### 2.10. Detection of Biofilm Formation in Flow Conditions by a BioFlux System

The BioFlux 1000 system (Fluxion Biosciences, Oakland, CA, USA) was used to detect dynamic biofilm formation in shear flow conditions. First, the microfluidic channels of a BioFlux 48-well plate were primed with 37 °C pre-warmed TSBG from the inlet wells. After the removal of the excess medium in the inlet wells [76,77,78], the mid-log phase *S. aureus* FPR3757 cells that were pre-cultured in TSBG (~10^8^ colony forming unit per mL) were flown into the channels and incubated for 1 h. After bacterial primary attachment, tilmicosin and oxacillin were added into the culture medium separately (16 μg/mL and 1 μg/mL, respectively) or in combination (8 μg/mL oxacillin and 0.5 μg/mL tilmicosin), then flowed at a stress of 0.15 dyn/cm^2^. Bacterial biofilm development was automatically monitored for 16 h with BioFlux Montage software 2.3 by acquiring the bright-field images at 10 min intervals [79,80,81].

### 2.11. Statistical Analysis

Student’s *t*-test was applied for data comparison. A *p*-value less than 0.05 was considered statistically significant.

## 3. Results

### 3.1. Effect of arlRS Knockout in Biofilm Formation in Multiple Staphylococcal Strains

**Figure 1:**
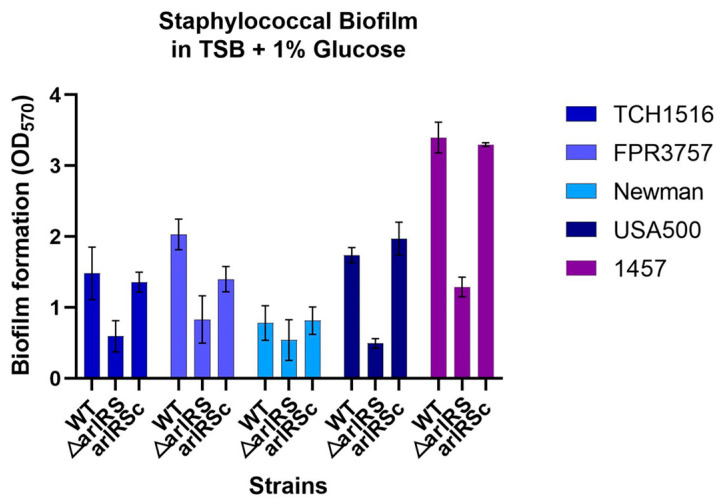
Impact of *arlRS* knockout on biofilm production of *S. aureus* and *S. epidermidis* strains in vitro. Biofilms produced by the *arlRS* knockout mutants and complementation strains of the MRSA TCH1516, FPR3757 and USA500 strains; the MSSA Newman strain; and *S. epidermidis* 1457 strain in TSBG in 96-well tissue-culture-treated polystyrene microplates for 24 h were compared with their parental strains.

To confirm the important role of *arlRS* in biofilm development in *S. epidermidis* and *S. aureus*, in vitro biofilm-forming abilities of the newly constructed *arlRS* knockout mutants of the MRSA strain USA300 FPR3757 and the MSSA strain Newman; the corresponding *arlRS* complementation strains (Table 1); and the previously constructed *arlRS* mutants of USA300 TCH1516, USA500 2395 and 1457 strains, were assessed by the classic microtiter plate assay. It showed that the biofilm formation in the *arlRS* mutants of TCH1516, FPR3757, USA500 and 1457 all decreased dramatically (OD_570_ = 0.59 ± 0.22, 0.83 ± 0.33, 0.49 ± 0.07, 1.29 ± 0.14) compared to their wild-type counterpart (OD_570_ = 1.48 ± 0.37, 2.03 ± 0.22, 1.73 ± 0.11, 3.40 ± 0.22) (Figure 1). Newman showed a weak biofilm formation (OD_570_ = 0.78 ± 0.24), and its *arlRS* mutant formed a mildly decreased biofilm (OD_570_ = 0.54 ± 0.29) with no significance. Complementation of *arlRS* in each of the *arlRS* mutants restored biofilm-forming abilities to almost the wild-type level.

### 3.2. Inhibitory Effect of Tilmicosin on ArlS Activity

**Figure 2:**
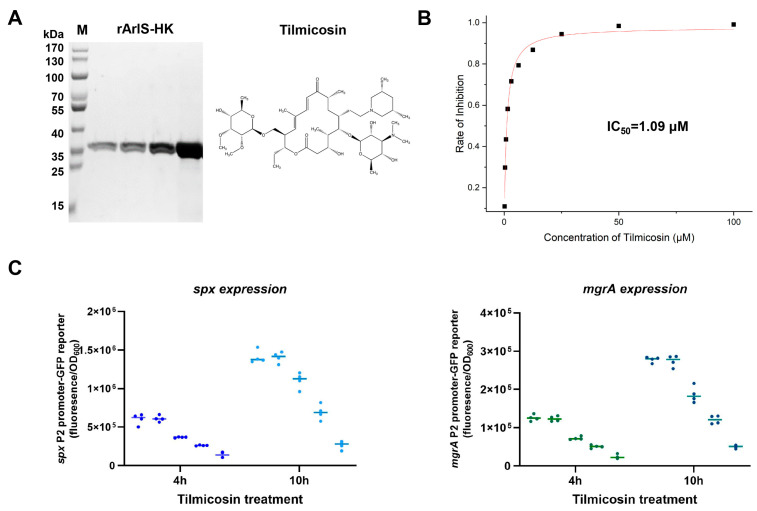
Inhibition of ArlS activity by tilmicosin: (**A**) the purified ArlSHK and the chemical structure of its kinase inhibitor tilmicosin; (**B**) dose-dependent inhibition of tilmicosin on the ArlSHK kinase activity detected by the Kinase-Glo(R) Luminescent Assay; (**C**) the effect of tilmicosin on ArlS activity in *S. aureus* at 4 h and 10 h, indicated by the *spx* P2 promoter and *mgrA* P2 promoter-GFP reporter systems (in each panel, from left to right: mock, 0.1% DMSO, 0.75 μM tilmicosin, 1.5 μM tilmicosin, Δ*arlRS* control).

As ArlRS is vital for biofilm development regulation, the cytoplasmic part of histidine kinase ArlS (ArlSHK) was purified and used for screening compounds that can inhibit its ability to hydrolyze ATP, thereby blocking signal transduction. The purified protein had an approximate molecular weight (MW) of 37 kDa (Figure 2A), and its kinase activity was confirmed (Figure S1). After primary drug screening, we found that tilmicosin (MW = 869.147 g/mol) at a concentration of 25 μM can inhibit the ArlSHK (2 μg) kinase activity by 95% and that its inhibitory effect was in a dose-dependent manner (Figure 2B). The data were further analyzed by using the sigmoidal fit module, and it showed that the half-maximal inhibitory concentration of tilmicosin was 1.09 μΜ. Tilmicosin had antibacterial activity, and its MIC was 3.1 μΜ (~2.7 μg/mL) in all of the MRSA and MSSA strains used in this study.

To investigate the effect of tilmicosin on ArlS activity and its subsequent regulation in *S. aureus*, two ArlRS regulon genes, *spx* and *mgrA*, which have been proven to be directly modulated by ArlRS [44,82,83,84,85], were chosen to serve as indicators for ArlRS regulation. The P2 promoters of the *spx* and *mgrA* were used for constructing promoter-green fluorescence reporter systems in the USA300 FPR3757 strain and its Δ*arlRS* mutant. Tilmicosin was added to the mid-exponential phase culture of the reporter strains of FPR3757 at a concentration of 0.75 μM or 1.5 μM. The DMSO treatment served as a negative control, and the reporter strains of Δ*arlRS* were used as low *spx* and *mgrA* expression controls. After further cultivation for 4 h and 10 h, the 0.75 μM tilmicosin group showed obviously lower *spx* and *mgrA* promoter activities than the mock and DMSO groups. Both promoter activities were even lower in the 1.5 μM tilmicosin group (Figure 2C).

### 3.3. Effect of Tilmicosin on Static Biofilms Formation of the Staphylococcal Strains

**Figure 3:**
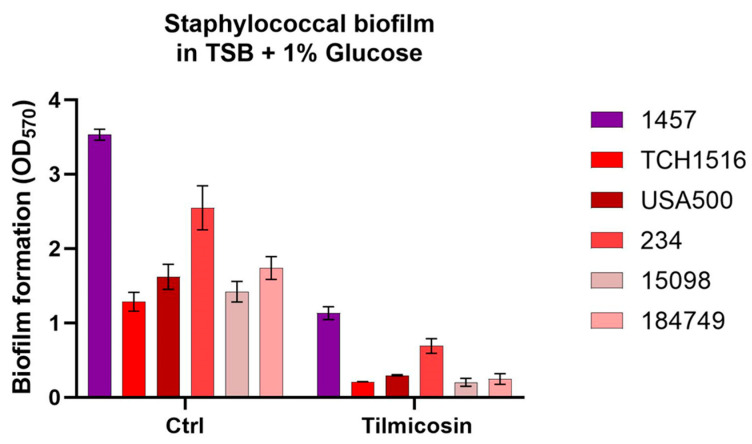
Inhibition of static staphylococcal biofilm production in vitro by tilmicosin. Effect of tilmicosin at a 1/4 of MIC on biofilms formed by the MRSA TCH1516, FPR3757 and USA500 strains; two clinical MRSA isolates (234 and 15,098); and the *S. epidermidis* 1457 strain were semi-quantitatively determined as described above.

To detect the activities of tilmicosin against *S. aureus* and *S. epidermidis* biofilms formation in static condition, a microtiter plate biofilm assay was performed. It showed that tilmicosin at 1/4 of MIC (0.78 μΜ or 0.67 μg/mL) inhibited MRSA biofilm formation by 72.9–85.8% and reduced *S. epidermidis* 1457 biofilm formation by 67.9% (Figure 3).

**Figure 4:**
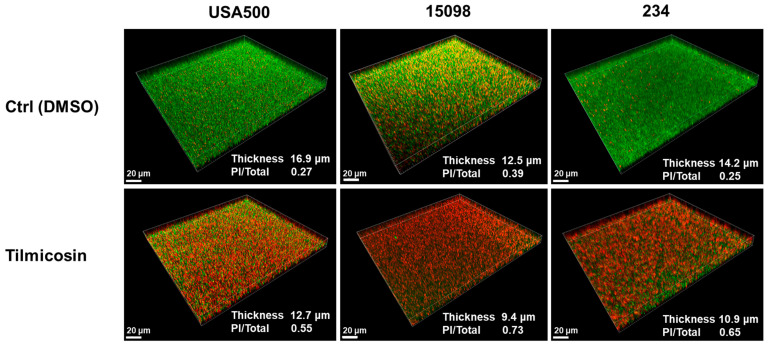
The impact of tilmicosin on MRSA young biofilms. The 1 μg/mL of tilmicosin or 0.1% DMSO (control) was added into pre-formed 6 h young biofilms of the MRSA strains USA500, 15,098 and 234 in TBSG in fluorodishes. After additional 18 h incubation, the biofilms remaining on the glass bottom were stained with live/dead staining kit. CLSM was performed to acquire fluoresent images, measure the biofilm thickness and analyze cell viability.

The impact of tilmicosin on cell viability in young biofilms of MRSA strains was further investigated by CLSM. The young biofilms (6 h) formed by the strains USA500, 15,098 and 234 in TBSG in fluorodishes were treated with 1 μg/mL (1/3 MIC) tilmicosin or 0.1% DMSO for 18 h. It showed that in the control group (0.1% DMSO), the average thickness of biofilm in each MRSA strain was 16.9, 12.5 and 14.2 μm, respectively, while tilmicosin treatment reduced the average thickness of biofilm to 12.7, 9.4 and 10.9 μm, respectively. Furthermore, the proportion of dead cells in the control group was 0.27, 0.39 and 0.25, respectively, while in the tilmicosin-treated biofilm, it was increased to 0.55, 0.73 and 0.65, respectively, indicated with a ratio of the PI intensity value and corresponding total (i.e., PI + SYTO9) intensity value (Figure 4).

### 3.4. Effect of Tilmicosin on MRSA Biofilm Formation in a Flow Condition

As ArlRS regulates oxacillin susceptibility in MRSA, we detected whether tilmicosin showed a synergistic effect with oxacillin against TCH1516, FPR3757, USA500, 234 and 15,098 strains in the planktonic state. The checkerboard method was performed to calculate the FICI. Although the FICI varied in those MRSA strains (0.25~0.5), the results all indicated a synergism of tilmicosin and oxacillin.

**Figure 5:**
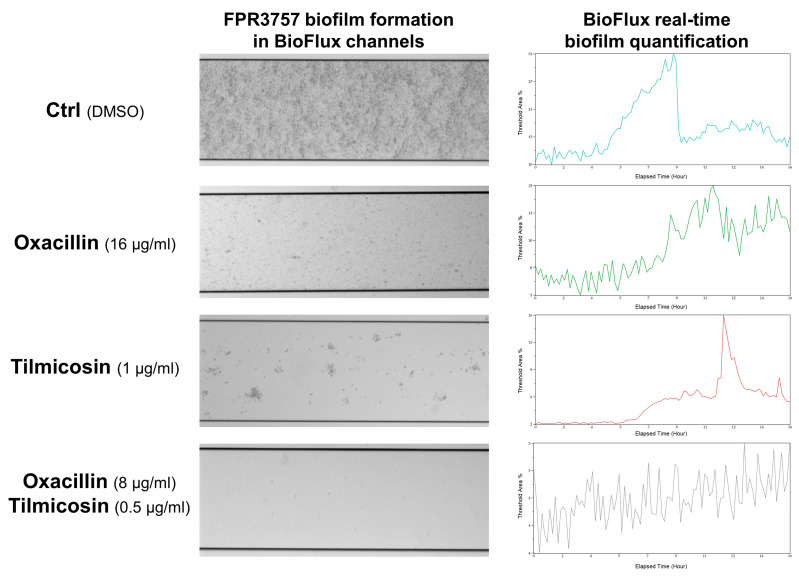
Inhibition of biofilm development of the MRSA strain USA300 FPR3757 by tilmicosin under a flowing condition (BioFlux 1000 system). The 16 h biofilm formed in the channels was photographed (**Left panel**). The amount of biomass was monitored and analyzed with BioFlux Montage software 2.3 in real time (**Right panel**).

The individual effect of tilmicosin and the combination effect of tilmicosin/oxacillin on dynamic biofilm development were further determined by using a BioFlux 1000 system. The USA300 FPR3757 cells in TSBG were seeded in the BioFlux microfluidic channels and incubated for 1 h for primary attachment, then fresh TSBG containing ~1/3 MIC of tilmicosin, ~1/4 MIC of oxacillin and a combination (~1/6 MIC of tilmicosin with ~1/8 MIC of oxacillin) were flowed into separate channels at a 0.15 dyn/cm^2^ stress. After real-time monitoring for another 16 h, a series of images were obtained, and continuous biomass was analyzed with BioFlux Montage software 2.3. In the 0.1% DMSO treatment control group, FPR3757 cells adhered tightly to the bottom of the channel (Figure 5, left panel) and gradually increased its biofilm biomass, which reached a peak at 8.5 h (Figure 5, right panel) and occupied ~33% threshold areas. In the channels treated with an individual agent (1 μg/mL tilmicosin; 16 μg/mL oxacillin), biofilm biomass showed a moderate decrease with delayed peaks at about 11.5 h, which occupied 16% and 15% threshold areas, respectively. In the drug combination group, it showed significant inhibition of biofilm formation (4~5% threshold areas), and no obvious peak was observed.

### 3.5. Effect of Tilmicosin on MRSA Mature Biofilms and Embedded Cells Viability

CLSM showed that mature biofilm (24 h) of the FPR3757 strain after 0.1% DMSO treatment had an average thickness of 13.6 μm and maintained the intact structure, while after 3 × MIC tilmicosin treatment, it not only showed a reduction in average thickness to 10.8 μm but also displayed a ruined structure. As a control, 8 μg/mL vancomycin treatment resulted in an increased biofilm thickness of 18.9 μm. Moreover, the proportion of dead bacterial cells in the DMSO and vancomycin groups was 0.28 and 0.41, respectively, while it was increased to 0.69 in the tilmicosin-treated mature biofilm.

## 4. Discussion

Two-component signal transduction systems are signal sensing-responding systems in bacteria, regulating their antimicrobial resistance, adaption and survival in various environments, as well as virulence in hosts. To date, no TCST homolog has been identified in humans. Thus, TCSTs have been recognized as novel antibacterial targets.

In a TCST, histidine kinase (HK), which contains a conserved substrate binding pocket, has been considered a good candidate for drug design. In this work, we used the catalytic and ATP-binding (CA) domain of ArlS HK for screening compounds that can inhibit the ATPase enzyme activity. The inhibitors block HK activity to hydrolyze ATP and subsequently hinder self-phosphorylation of the protein, thereby interfering with the transfer of the phosphate group to its cognate response regulator (RR), which is involved in transcriptional regulation. This strategy has been demonstrated to be feasible and effective. Previous studies in our laboratory have discovered novel compounds targeting *S. epidermidis* essential YycG histidine kinase CA domain, which have potent inhibitory effects on staphylococcal growth and biofilm formation [74,86,87,88,89,90,91,92,93]. Recently, there has been inspiring progress on the development of novel inhibitors against several other HKs, including *S. aureus* VraS (involved in cell call-targeting antibiotics susceptibility) [94], AgrC (important for quorum sensing and virulence regulation) [95], etc. In addition, the extracellular domain of HK is responsible for sensing environment signals, so it may also serve as a potential target for screening inhibitory peptides or monoclonal antibodies (mAbs) that can block signal recognition [96,97]. Our laboratory has made efforts to develop the mAbs that bind to the epitopes in the extracellular domain of YycG. The mAbs show good anti-biofilm activities [98]. Although HK attracts the most attention for inhibitor development, there are also several reports about inhibitors targeting RR protein. RR typically contains a conserved receiver (REC) domain and an effector domain (DNA binding). REC functions as a phosphorylation-mediated switch within RR and controls the activity of the effector domain that elicits output responses. In addition to previously reported Walrycin B targeting *S. aureus* RR WalR [99], SaeR has recently been found to be a novel target for antivirulence therapy [100].

As a TCST that is unique in staphylococci, ArlRS has been proven to have vital functions in regulating *S. epidermidis* and *S. aureus* biofilm production, modulating *S. aureus* virulence and affecting antibiotic resistance in MRSA strains [101]. These findings indicate that ArlRS is a promising target for screening inhibitors that may eradicate young/mature biofilms, retard virulence and break antimicrobial resistance. We found that oritavancin has an inhibitory effect against ArlS kinase activity with an IC_50_ of 5.47 μM, and it has a synergistic effect with oxacillin. Recently, Kwiecinski et al. reported that 3,4′-dimethoxyflavone directly inhibits ArlS autophosphorylation and decreases the severity of MRSA infection in mice [102]. In this study, our group found that tilmicosin can strongly inhibit the ArlSHK kinase activity, with an IC_50_ of 1.09 μM (about 1/5 of the oritavancin IC_50_), indicating tilmicosin has more potent inhibition than oritavancin.

**Figure 6:**
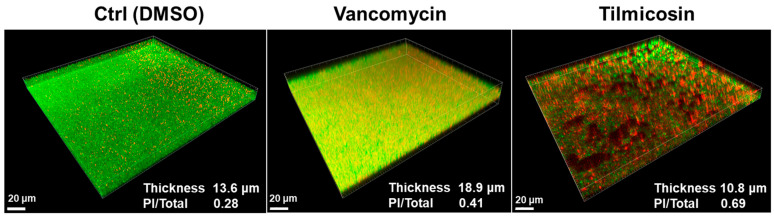
Effect of tilmicosin on MRSA mature biofilms. The 8 μg/mL of tilmicosin, 8 μg/mL of vancomycin or 0.1% DMSO (control) was added into pre-formed 24 h mature biofilm of the MRSA strains USA300 FPR3757 in fluorodishes. After additional 24 h incubation and live/dead staining, the biofilm thickness and fluorescence intensities were measured as described above.

As a macrolide, tilmicosin has an antibacterial activity by inhibiting protein synthesis. The MIC of tilmicosin against MRSA strains obtained in this work was 3.1 μM (about 2.7 μg/mL), which was similar to that in a previous report [103]. Our work shows that besides its known function, it also can inhibit ArlSHK kinase activity and subsequently interfere with ArlR regulation, suggesting another role against staphylococci, including MRSA. In a previous study, we demonstrated that the transcriptional regulator gene *spx* was under the direct control of the ArlRS system [44]. Crosby et al. confirmed our finding and further showed that the P2 promoter of *spx* contained a consensus motif that was recognized by ArlR [85]. A series of studies in their group proved that the P2 promoter of another gene *mgrA* is also directly modulated by ArlRS [82,83,84]. Thus, the P2 promoters of *spx* and *mgrA* were chosen for constructing transcription reporter systems that indicate ArlRS regulation. As a result, both *spx* and *mgrA* P2 promoters show low activities in tilmicosin treatment groups at 4 h and 10 h, suggesting a strong inhibition of ArlS activity by tilmicosin. Considering the importance of ArlRS in modulating oxacillin resistance and biofilm development, this may explain why tilmicosin shows synergism with oxacillin not only against MRSA proliferation in planktonic state but also against MRSA biofilm formation in flow condition. Furthermore, CLSM observation suggests that tilmicosin treatment disrupts mature *S. aureus* biofilms (Figure 6) and may subsequently facilitate its bactericidal activity against embedded MRSA cells (indicated by a significant increase in propidium iodide intensities). Overall, tilmicosin is a potent antibacterial and anti-biofilm agent that may be used in the treatment of acute *S. aureus* infections and biofilm-associated infections in animals. Additionally, it has a potential to serve as a lead compound for future structural modifications that may broaden its applications to humans.

## 5. Conclusions

ArlRS is important for biofilm formation in different strains of *S. aureus* and *S. epidermidis*. Tilmicosin has a potent inhibition on ArlSHK kinase activity and biofilm formation in both static and flow conditions. Tilmicosin can disrupt MRSA mature biofilms and kill embedded MRSA cells. Furthermore, tilmicosin has a synergy effect with oxacillin against MRSA proliferation and biofilm formation.

## Figures and Tables

**Table 1:** The staphylococcal strains and plasmids constructed in this work.

Staphylococcal Strains/Plasmids	Information
Strains	
FPR3757Δ*arlRS*	an *arlRS* knockout strain of the MRSA USA300 FPR3757
NewmanΔ*arlRS*	an *arlRS* knockout strain of the MSSA Newman
*arlRSc* (FPR3757)	an *arlRS* complementation strain of the FPR3757Δ*arlRS*
*arlRSc* (USA500)	an *arlRS* complementation strain of the USA500Δ*arlRS*
*arlRSc* (Newman)	an *arlRS* complementation strain of the NewmanΔ*arlRS*
FPR3757-P*mgrA-P2*	a *mgrA* promoter-reporter strain by transforming the USA300 FPR3757 with the plasmid pCM29-*mgrA-P2*
FPR3757-P*spx-P2*	a *spx* promoter-reporter strain by transforming the USA300 FPR3757 with the plasmid pCM29-*spx-P2*
Δ*arlRS*::P*mgrA-P2*	FPR3757Δ*arlRS* harboring pCM-*mgrA-P2*
Δ*arlRS*::P*spx-P2*	FPR3757Δ*arlRS* harboring pCM-*spx-P2*
Plasmids	
pCM-*spx-P2*	pCM29 modified by replacing its GFP promoter with *spx* P2 promoter
pCM-*mgrA-P2*	pCM29 modified by replacing its GFP promoter with *mgrA* P2 promoter
pRB-500*arlRS*	pRB475 carrying the *arlR* and *arlS* genes of USA500
pRB-NM*arlRS*	pRB475 carrying the *arlR* and *arlS* genes of Newman
pETMG-*arlSHK*	the *arlS* gene histidine kinase region cloned into pETMG

## Data Availability

Data are contained within the article or supplementary materials.

## Supplementary Materials

The following supporting information can be downloaded at: https://www.mdpi.com/article/10.3390/microorganisms12020256/s1, Figure S1: Primers used in this study; Table S1: Detection of ATPase activity of the recombinant ArlSHK.
