# Supplementary material for: The Staphylococcus aureus ArlS Kinase Inhibitor Tilmicosin Has Potent Anti-Biofilm Activity in Both Static and Flow Conditions
Source: Microorganisms. 2024 Jan 25;12(2):256. doi: 10.3390/microorganisms12020256 (PMC10891534; doi:10.3390/microorganisms12020256)
Supplement: Supplementary file 1 [file microorganisms-12-00256-s001.zip › Figure S1.pdf]

Figure S1

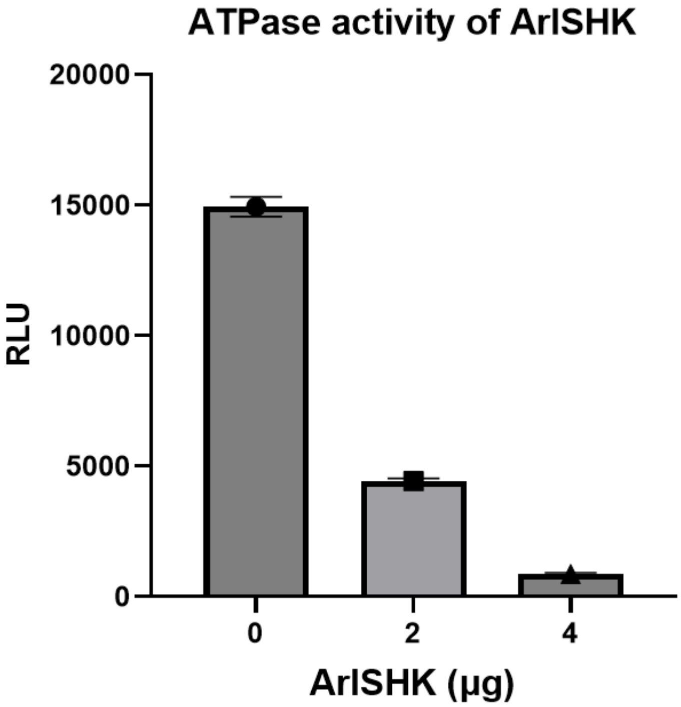

Figure S1. Detection of ATPase activity of the recombinant ArlSHK

The Kinase-Glo(R) Luminescent Kinase Assay (Promega) was using to detect ATPase activity of the purified ArlSHK. Briefly, 2 μg or 4 μg purified ArlSHK was mixed with 4 μM ATP in 50 μl reaction buffer, then incubated for 30 min at room temperature. Afterwards, the reaction mixture was added with 50 μl Kinase-Glo(R) Reagent and kept at room temperature for 10 min. The luminescence (RLU) indicating remaining ATP was recorded by a Victor X5 reader.
