# Supplementary material for: The Staphylococcus aureus ArlS Kinase Inhibitor Tilmicosin Has Potent Anti-Biofilm Activity in Both Static and Flow Conditions
Source: Microorganisms. 2024 Jan 25;12(2):256. doi: 10.3390/microorganisms12020256 (PMC10891534; doi:10.3390/microorganisms12020256)
Supplement: Supplementary file 1 [file microorganisms-12-00256-s001.zip › Table S1.pdf]

Table S1. Primers used in this study

Primers	Sequences (5'-3')	Restriction Site
**For construction of the promoter-GFP reporter plasmids**		
SA-*spx*-P2-pro-F	CTCGCTAGCGTGTAGAAACTTATCTGAAAAATTAGG	NheI
SA-*spx*-P2-pro-R	CTCGGATCCTCATTGCTTAAAATTTAGTTATAGATCAAG	BamHI
SA-*mgrA*-P2-pro-F	CACGCTAGCGATCGCAACAAACACAACC	NheI
SA-*mgrA*-P2-pro-R	CACGGATCCGTTACTGACACATTATATTAGTGA	BamHI
**For construction of the *arlRS* complementation plasmids**		
SA-*arlRSc*-R	GTTGGATCCTGACTTTGATTGACGTCTCAGT	BamHI
SA-*arlRSc*-F	GTTGTCGACTGTGTGAAATGTTTAAACTACGGT	SalI
**For expression of the recombinant cytoplasmic domain of ArlS protein**		
arlSHK-F	TCGGGATCCATTCGACGAGATGGTTTTC	BamHI
arlSHK-R	TCGCTCGAGTAAAATATGATTTTAAACGTTGTTCCT	XhoI
